# The gas stove-childhood asthma kerfuffle: A teaching opportunity

**DOI:** 10.1016/j.gloepi.2023.100104

**Published:** 2023-03-29

**Authors:** Louis Anthony Cox

**Affiliations:** Cox Associates, MoirAI, Entanglement, University of Colorado, 503 N. Franklin Street, Denver, CO 80218, USA

**Keywords:** Indoor air pollution, Gas stoves, Childhood asthma, Questionable research practices

## Abstract

Several recent news stories have alarmed many politicians and members of the public by reporting that indoor air pollution from gas stoves causes about 13% of childhood asthma in the United States. Research on the reproducibility and trustworthiness of epidemiological risk assessments has identified a number of common questionable research practices (QRPs) that should be avoided to draw sound causal conclusions from epidemiological data. Examples of such QRPs include claiming causation without using study designs or data analyses that allow valid causal inferences; generalizing or transporting risk estimates based on data for specific populations, time periods, and locations to different ones without accounting for differences in the study and target populations; claiming causation without discussing or quantitatively correcting for confounding, external validity bias, or other biases; and not mentioning or resolving contradictory evidence. We examine the recently estimated gas stove-childhood asthma associations from the perspective of these QRPs and conclude that it exemplifies all of them. The quantitative claim that about 13% of childhood asthma in the United States could be prevented by reducing exposure to gas stove pollution is not supported by the data collected or by the measures of association (Population Attributable Fractions) used to analyze the data. The qualitative finding that reducing exposure to gas stove pollution would reduce the burden of childhood asthma in the United States has no demonstrated validity. Systematically checking how and whether QRPs have been addressed before reporting or responding to claims that everyday exposures cause substantial harm to health might reduce social amplification of perceived risks based on QRPs and help to improve the credibility and trustworthiness of published epidemiological risk assessments.

## Highlights

• Recent news stories have reported that gas stove emissions cause about 13% of childhood asthma in the United States.
• Previous literature identifies questionable research practices (QRPs) to avoid in drawing sound causal conclusions.
• Key QRPs include claming causality without doing causal analysis or collecting data that supports vaid causal inferences.
• The claims about gas stoves causing childhood asthma exemplify previously identified QRPs.
• These claims are not supported by the data collected or the analyses performed.  Checking QRPs can improve risk analyses.

## 1 Introduction

In early 2023, several high-profile news stories stirred considerable discussion and dismay by reporting that use of gas stoves was linked to an increased risk of childhood asthma and that mitigating this exposure would prevent about 13% of childhood asthma cases in the United States. For example, the *Washington Post* ran a headline stating that “Gas stove pollution causes 12.7% of childhood asthma” and noted that some regulators were considering a ban on new gas stoves [8]. For policymakers and members of the public who trusted this news, the necessity of choosing between children's health and gas cooking loomed. For more skeptical observers, the possibility arose that Ioannidis's 2005 provocative dictum that “most published research findings are false” (Ioannidis, 2005) was about to be vividly illustrated again. For methodologists concerned with making and keeping epidemiology-based public health risk assessment a credible science, the news stories raise the usual important challenges of evaluating an important policy-relevant causal claim: is the claimed preventability of childhood asthma based on sound analysis and interpretation of relevant data? This note reviews questions that epidemiologists, reporters, and members of the public can ask to help critically evaluate the studies behind such news stories and to help distinguish between trustworthy and untrustworthy claims of adverse health effects from common exposures.

## 2 Underlying study findings and causal interpretations

The occasion for the 2023 news stories was an article by Gruenwald et al. [6] that applied population attributable fraction (PAF) calculations to estimated gas stove prevalence and current childhood asthma data in the US based on a previously published measure of statistical association (Odds Ratio = 1.34) estimated in a 2013 meta-analyses by Lin et al. [10]. The Gruenwald et al. paper is brief (just over 3 pages including abstract and references), as it simply applies pre-existing PAF estimates from Lin et al. to gas stove usage data for the US. Its final paragraph, which generated many headlines, states that “In conclusion, 12.7% of current childhood asthma nationwide is attributed to gas stove use, which is similar to the childhood asthma burden attributed to secondhand smoke. Gas stove usage should be considered in multi-faceted asthma prevention approaches. Given that this exposure is preventable, our study demonstrates that known mitigation strategies will lessen childhood asthma burden from gas stoves, particularly in states with elevated PAFs.”

The meta-analysis paper of Lin et al. et al. is more substantial (13 pages). It explains that “We extracted the association between indoor NO2 (and gas cooking) and childhood asthma and wheeze from population studies published up to 31 March 2013.” It concludes that “Household gas cooking is associated with increased odds of current asthma and lifetime asthma in children. The risk of overall asthma in children with gas cooking exposure [more specifically, the summary odds ratio from random effects meta-analysis of 41 studies] was 1.32 (95% confidence interval, 1.18–1.48).” Lin et al. interpreted this odds ratio as follows: “This meta-analysis provides quantitative evidence that, in children, gas cooking increases the risk of asthma.”

## 3 Critical analysis: What questions should we ask?

In assessing the causal claims of studies that estimate substantial adverse health effects from everyday exposures, it is helpful to stimulate and organize critical thinking by asking how they have dealt with the most prevalent questionable research practices (QRPs) that commonly threaten the validity of epidemiologically based claims about health risks caused by exposures. Studies that list common QRPs, estimate their prevalence in published articles, and recommend ways to correct or forestall them have increased in the decades following the controversial but influential essay of Ioannidis [7]. A useful recent compendium by Gerrits et al. [5] includes the following QRPs to avoid when making causal claims about adverse health effects of exposures:

• Hypotheses contain unsupported causality
• Generalization to different populations (or different time periods, locations, or settings)
• Causation claimed without discussing bias
• Causation claimed without appropriate design
• Contradictory evidence is not mentioned

The following paragraphs examine the studies of Gruenwald et al. and Lin et al. from these perspectives.

### 3.1 Were study designs and data appropriate for drawing valid causal inferences?

A striking feature of both papers is that they only analyze statistical *associations* (odds ratios and PAFs) in observational data. These associations are limited to showing that *differences* in risk are associated with *differences* in exposures. Both papers nonetheless draw *causal* conclusions about how *changes* in exposure would *change* risk – albeit without using study designs, data, or analyses that examine changes in either exposure or risk. Thus, Gruenwald et al. state that “Our study demonstrates that known mitigation strategies will *lessen* childhood asthma burden from gas stoves” and Lin et al. state that “In children, gas cooking *increases* the risk of asthma” (emphases added).

These interpretations exemplify a QRP referred to by Gerrits et al. [5] as “Inappropriate use of evidence,” in this case to draw conclusions about changes in risk from evidence that does not directly address changes in risk. This violates a basic tenet of sound causal analysis in epidemiology: association should not be conflated with causation, differences should not be conflated with changes [11], and *risks attributed to an exposure based on statistical associations of differences in risk with differences in exposure do not predict how or whether changing exposure would cause risk to change* [2]*.* For example, Pearl [11] emphasized the “basic distinction” between (a) statistical analyses of data on observed levels of variables (termed associational analyses); and (b) causal analyses capable of predicting the effects of interventions. He stated that “Causal analysis goes one step further [than associational analysis]; its aim is to infer not only beliefs or probabilities under static conditions, but also the dynamics of beliefs under changing conditions, for example, changes induced by treatments or external interventions. This distinction implies that causal and associational concepts do not mix. … [T]here is nothing in a distribution function to tell us how that distribution would differ if external conditions were to change.” The underlying studies in the Lin et al. meta-analysis were not intervention studies or quasi-experiments with control groups designed to support valid causal inferences. They were associational studies of differences in childhood asthma rates among households with different levels of exposure to gas stoves (and perhaps other variables, as detailed later in discussing confounding). The “basic distinction” between associational and causal analyses highlighted by Pearl implies that such studies do not support valid causal inferences about how much or whether interventions that change exposure to gas stoves would change childhood asthma. From this perspective, the assertions that “Our study demonstrates that known mitigation strategies will lessen childhood asthma burden from gas stoves” by Gruenwald et al. and that “In children, gas cooking increases the risk of asthma” by Lin et al. go beyond what the data analyzed can show. It may or may not be true that gas cooking increases the risk of childhood asthma, but the answer is not revealed by the odds ratios. The study of Gruenwald et al. based on odds ratios does *not* demonstrate the effects of mitigations, as such effects are not addressed in calculating the odds ratios. These assertions are examples of the QRP “Causation claimed without appropriate design.”

### 3.2 Were appropriate causal analysis methods used?

Suppose that appropriate data and study designs for drawing valid causal inferences had been used, e.g., by comparing the changes in the prevalence of childhood asthma (or the frequency of childhood asthma attacks) in a population before and after an intervention using a difference-in-differences (DID) design with well-designed control groups and with all the assumptions of the DID analysis satisfied [12]. Then estimates of the causal impact of the intervention could be achieved using appropriate statistical analyses of the data, specifically, DID regression analysis (ibid). But even in this idealized setting, PAF would not be a valid measure of the causal impact of an intervention because it does not account for the effects of other covariates (e.g., socioeconomic variables or housing quality) that may affect asthma risk. The Gruenwald et al. paper treats PAF as a measure of *preventable* risk, stating that “The proportion of childhood asthma that could be theoretically prevented if gas stove use was not present (e.g., state-specific PAFs) varied by state (Illinois = 21.1%; California = 20.1%; New York = 18.8%; Massachusetts = 15.4%; Pennsylvania = 13.5%). Our results quantify the US public health burden attributed to gas stove use and childhood asthma.” But the PAF is based only on a measure of association (specifically, relative risk). It is not a measure of preventability. In the presence of confounding, for example, a PAF may be as high as 100% even if the fraction of cases that would be prevented by removing exposure is 0% [3]. As cautioned by Counil [1], “PAFs are estimated based on strong assumptions and the calculations are data intensive, making them vulnerable to gaps in knowledge and data.” Typical assumptions are that exposure is causally related to disease; that data used to estimate the PAF in the target population are valid and accurately measured; that the exposure being studied is independent of other risk factors for the disease; that the exposure does not cause comorbidities or outcomes that could affect the estimates of the PAF; and that the (assumed) causal relationship between exposure and response is stable over time. Applying previously estimated PAFs from one population (e.g., a mix of European and North American studies from the years prior to 2013) to a new target population (e.g., the current population of one of the above US states) without verifying these strong assumptions leads to risk estimates of unknown validity.

This limitation on the data analysis side matches the limitation noted above on the study design side: the analysis done, like the data collected, does not justify the causal conclusion that “Our study demonstrates that known mitigation strategies will lessen childhood asthma burden from gas stoves” [6]. If the PAF is positive because of unaccounted-for differences in covariates between households with and without gas cooking, then this would illustrate the QRPs of “Causation claimed without discussing bias” (specifically bias due to omitted confounders) and “Hypotheses contain unsupported causality” where the null hypothesis tested and rejected is the non-causal one that PAF = 0 but the hypothesis accepted in stating the conclusion contains an unsupported causal claim that reducing exposure to gas stoves would reduce childhood asthma. These are treated as equivalent propositions but they are not: PAF could be positive even if reducing exposure has no effect on asthma risk. This is discussed further next in considering confounding.

### 3.3 Were confounding and residual confounding accounted for quantitatively?

Neither Gruenwald et al. nor Lin et al. provide quantitative adjustments for confounding. Lin et al. note that “*Residual confounding by (unmeasured) factors that are associated with gas cooking might be another explanation for our finding* of an association between asthma and gas cooking.” (Emphasis added.) Gruenwald et al. do not mention this possibility. Lin et al. then dismiss residual confounding without further analysis, opining that “However, this is not very likely as we used effect estimates from the included studies which were almost always adjusted for known determinants of childhood asthma.” This argument appears to misunderstand the nature of residual confounding, which is defined as confounding that remains even *after* such adjustments have been made, e.g., due to the use of broad categories to summarize quantitative variables or due to error in measuring adjustment variables. Lin et al. themselves used several such broad categorizations of continuous variables, e.g., dichotomizing the proportion of gas cooking as at least 30% or <30%; dichotomizing study publication year as before 2000 or not; and categorizing age as 6–10 years, <6 years, or >10 years. Moreover, examining the individual studies that Lin et al. rely on shows that they do *not* rule out confounding as a plausible explanation for epidemiological associations between asthma and gas cooking. For example, the first study cited by Lin et al., a study by Belanger and Triche [13], states that “Gas stove use is particularly common in central cities; in the United States, inner cities are associated with high rates of poverty and substandard housing. In rural areas, the use of secondary heating devices (kerosene heaters, unvented gas) may be a means to reduce heating costs among low-income families. Thus*, it is possible that gas stoves and portable heating devices are markers for poverty and substandard housing, and this confounds any association with asthma*.” (Emphasis added.) Neither Lin et al. nor Gruenwald et al. mention this possibility or offer quantitative adjustments for confounding by poverty and substandard housing (or other potential confounders). Thus, an unknown proportion (perhaps 100%) of the association they describe may be due to uncontrolled confounding.

Consistent with this possibility, Belanger and Triche note that “A case control study in Baltimore, MD, specifically looked at exposure to gas stoves among low-income children in the United States. The investigators recruited 150 asthma cases and 150 controls to investigate factors associated with asthma development. All children lived in the inner city and high percentages were from low-income families (88% Medicaid) and were African American (91%). *Asthma was not associated with either gas stove exposure or heating systems among these children*.” (Emphasis added.) Lin et al. did not mention that controlling (via stratification) for potential confounders such as low income caused the estimated statistical “effect” (i.e., association) of gas stoves on childhood asthma to disappear in at least some of the studies they cite in reaching their conclusion that “In children, gas cooking increases the risk of asthma”. This illustrates the very common QRP “Contradictory evidence is not mentioned.”

### 3.4 Was the heterogeneity of associations modeled and adjusted for in transporting effects estimates?

Gruenwald et al. state that “We combined effect sizes for North America and Europe given the similarities in housing characteristics and gas-stove usage patterns across these geographies.” However, the data of Lin et al. show substantial heterogeneity in the estimated “effects sizes” (i.e., associations) in the underlying studies. For example, Fig. 2 of Lin et al. shows odds ratios for gas cooking-current childhood asthma associations ranging from 0.69 to 2.28, with 3 of 11 studies reporting odds ratios <1. Lin et al., explain their approach to dealing with this unexplained heterogeneity as follows: “Due to the heterogeneity among studies which were performed independently by different researchers in different populations, pooled risk estimates were calculated by random-effect models with inverse-variance weights.” A single (“pooled”) odds ratio was estimated for the gas cooking-asthma association. This obscures the fact that different studies differed widely on the estimated “effect size” and even on whether it was positive (OR > 1) or negative (OR < 1). Using a single pooled estimate makes sense when all studies are estimating the same value with varying degrees of precision based on varying sample sizes, but not when the odds ratio being estimated truly differs across studies. If there is real heterogeneity, rather than just sampling variability, then it has been standard practice for many years in epidemiology to understand and adjust for it before estimating risk under new conditions, such as in the United States today rather than in Europe several decades ago.

Heterogeneity in odds ratios can arise if they depend on factors other than exposure such as population demographics, co-exposures, co-morbidities, or other covariates that have different distributions in different study populations. To adjust for these differences, conditional probability models for external validity (allowing for generalization and transportability of effects estimates across populations with different distributions of covariates) have been extensively developed in the past decade [4]. Neither Gruenwald et al. nor Lin et al. characterized the differences in covariates that might explain the heterogeneity in estimated odds ratios. Neither used transportability formulas to adjust the ORs and PAFs estimated from other countries, times, and settings to apply specifically to locations in the United States today. Although Lin et al. did note that “Almost half of the included studies were published before 2000. The estimated effects of gas cooking on asthma were higher in studies that were published before the year 2000,” Gruenwald et al. did not adjust for the year in projecting current (post-2020) risks for the United States. In terms of the QRPs listed by Gerrits et al., this corresponds to “Generalization to different time period” (and to different location, setting, and population) and also to “Causation claimed without discussing bias,” specifically, external validity bias. As noted by Degtiar and Rose [4], “both internal and external validity are necessary for unbiased estimates in a target population.” External validity bias can now be addressed by constructive methods, developed largely over the past decade, that include tests for the heterogeneity of treatment effects (HTE) and for differences between study and target populations as well as transportability formulas that adjust for differences in the distributions of covariates to allow data collected under one set of conditions (e.g., in Europe several decades ago) to be used to predict risk under a different set of conditions (e.g., in the US today) (ibid). The calculations of Gruenwald et al. did not make adjustments to prevent either internal validity biases (e.g., from uncontrolled confounding, as discussed above) or external validity biases.

## 4 Lessons and recommendations

Some suggested lessons and recommendations from the foregoing observations are as follows.

• The causal claims that “Our study demonstrates that known mitigation strategies will lessen childhood asthma burden from gas stoves” [6] and that “Gas stove pollution causes 12.7% of childhood asthma” [8] are not supported by the data analyzed because the underlying study designs and data do not address effects of mitigations or resulting changes in childhood asthma burden.
• These claims are also not supported by the analyses performed since these analyses only quantify measures of association (ORs and PAFs derived from relative risk ratios) but not measures of preventability or of the causal impact of exposures on asthma risk.
• Alternative plausible explanations for the reported statistical associations between gas stove cooking and childhood asthma, such as confounding by poverty and substandard housing (Belanger and Triche, 2008), were ignored.
• Differences between the times, populations, settings, and locations for which data were collected and the current US were ignored in extrapolating associations – many of them estimated in European countries before 2000 – to current US populations. Yet, the underlying meta-analysis of Lin et al. noted that associations were stronger in data collected before 2000 than in more recent data.
• Because of these limitations, the projections of Gruenwald et al. that about 13% of childhood asthma in the US could be prevented by reducing or eliminating gas stove emissions have no known validity. They are not supported by the data and analyses performed.
• These limitations follow peviously identified common patterns of prevalent questionable research practices (QRPs) that undermine the reliability and validity of much of the recent research literature in applied epidemiology [5].
• By insisting on routinely asking how (or whether) these QRPs have been addressed, research authors, journal reviewers and editors, reporters, politicians, and members of the public can help to judge (and document) the extent to which causal claims of adverse health effects from exposures are well supported by data and analyses.
• A widespread habit of QRP-checking might perhaps help all parties to improve the credibility and trustworthiness of published results by systematically identifying and downplaying claims that rely on QRPs.
• Exercising such critical thinking before broadcasting and responding to sensational claims about adverse health effects caused by everyday exposures might help to reduce the social amplification of risk [9] and encourage more responsible risk research and reporting.

Viewing announcements of health effects being “linked” to (i.e., associated with) various exposures as opportunities to apply critical thinking and to check the logical validity of causal claims against well-known QRPs may help to teach the value and spread understanding of sound, critical, epidemiological reasoning about causal claims and their policy implications.

## Declaration of Competing Interest

The authors declare that they have no known competing financial interests or personal relationships that could have appeared to influence the work reported in this paper.

## References

[1] Counil E. (2021 Dec). Contribution of causal factors to disease burden: how to interpret attributable fractions. Breathe (Sheff).

[2] Cox L.A. (2023 Jan). Re-assessing human mortality risks attributed to PM2.5-mediated effects of agricultural ammonia. Environ Res.

[3] Cox L.A. (2021). Toward practical causal epidemiology. *Global*. Epidemiology..

[4] Degtiar I., Rose S. (2023). A review of generalizability and transportability. Annu Rev Stat Applic.

[5] Gerrits R.G., Jansen T., Mulyanto J. (2019). Occurrence and nature of questionable research practices in the reporting of messages and conclusions in international scientific health services research publications: a structured assessment of publications authored by researchers in the Netherlands. BMJ Open.

[6] Gruenwald T., Seals B.A., Knibbs L.D., Hosgood H.D. (2022 Dec 21). Population attributable fraction of gas stoves and childhood asthma in the United States. Int J Environ Res Public Health.

[7] Ioannidis J.P. (2005 Aug). Why most published research findings are false. PLoS Med.

[8] Joselow M. (2023). @923. https://www.washingtonpost.com/politics/2023/01/06/gas-stove-pollution-causes-127-childhood-asthma-study-finds/.

[9] Kasperson R.E., Webler T., Ram B., Sutton J. (2022 Jul). The social amplification of risk framework: new perspectives. Risk Anal.

[10] Lin W., Brunekreef B., Gehring U. (2013 Dec). Meta-analysis of the effects of indoor nitrogen dioxide and gas cooking on asthma and wheeze in children. Int J Epidemiol.

[11] Pearl J. (2009). Causal inference in statistics: an overview. Stat Surv.

[12] Wing C., Simon K., Bello-Gomez R.A. (2018 Apr 1). Designing difference in difference studies: best practices for public health policy research. Annu Rev Public Health.

[13] Belanger K., Triche E.W. (2008). Indoor combustion and asthma. Immunol Allergy Clin North Am.

